# The effect of tinzaparin on biomarkers in FIGO stages III-IV ovarian cancer patients undergoing neoadjuvant chemotherapy – the TABANETOC trial: study protocol for a randomized clinical multicenter trial

**DOI:** 10.2340/1651-226X.2024.40207

**Published:** 2024-07-22

**Authors:** Anna Karlsson, Gabriel Lindahl, Anna-Clara Spetz Holm, Karin Bergmark, Pernilla Dahm Kähler, Boglarka Fekete, Ulrika Ottander, Charlotte Öfverman, Pernilla Israelsson, Laila Falknäs, Anders Rosenmüller, Malena Tiefenthal Thrane, Shefqet Halili, Tomas L. Lindahl, Maria C. Jenmalm, Preben Kjølhede

**Affiliations:** a Department of Obstetrics and Gynecology, Department of Biomedical and Clinical Sciences, Linköping University, Linköping, Sweden; b Department of Oncology, Department of Biomedical and Clinical Sciences, Linköping University, Linköping, Sweden; c Department of Oncology, Sahlgrenska University Hospital, Gothenburg, Sweden; d Department of Obstetrics and Gynecology, Sahlgrenska Academy at University of Gothenburg, Gothenburg, Sweden; e Department of Clinical Sciences, Obstetrics and Gynecology, Umeå University, Umeå, Sweden; f Department of Diagnostics and Intervention, Oncology, Umeå University, Umeå, Sweden; g Department of Obstetrics and Gynecology, Ryhov hospital, Jönköping, Sweden; h Department of Obstetrics and Gynecology, Västervik hospital, Västervik, Sweden; i Department of Obstetrics and Gynecology, Höglandssjukhuset, Eksjö, Sweden; j Department of Obstetrics and Gynecology, Värnamo hospital, Värnamo, Sweden; k Department of Biomedical and Clinical Sciences, Division of Clinical Chemistry and Pharmacology, Linköping University, Linköping, Sweden; l Division of Inflammation and Infection, Department of Biomedical and Clinical Sciences, Linköping University, Linköping, Sweden

**Keywords:** Clinical trial, neoadjuvant chemotherapy, ovarian cancer, tinzaparin

## Abstract

**Background:**

Tinzaparin, a low-molecular weight heparin (LMWH), has shown anti-neoplastic properties in animal models and in *in vitro* studies of human cancer cell lines. The reduction of CA-125 levels during neoadjuvant chemotherapy (NACT) in patients with epithelial ovarian cancer (EOC) co-varies with the prognosis; the larger the decrease in CA-125, the better the prognosis.

**Purpose:**

This study aims to evaluate the potential anti-neoplastic effects of tinzaparin by investigating changes in serum CA-125 levels in advanced EOC patients who receive NACT.

**Material and methods:**

This is an open randomized multicenter pilot trial. Forty patients with EOC selected to receive NACT will be randomized 1:1 to receive daily addition of tinzaparin or no tinzaparin. The processing and treatment of the patients will otherwise follow the recommendations in the Swedish National Guidelines for Ovarian Cancer. Before every cycle of chemotherapy, preoperatively, and 3 weeks after the last cycle of chemotherapy, a panel of biomarkers, including CA-125, will be measured.

**Patients:**

Inclusion criteria are women aged 18 years or older, World Health Organization performance status 0–1, histologically confirmed high-grade serous, endometrioid or clear cell EOC, International Federation of Gynecology and Obstetrics (FIGO) stages III-IV. In addition, a CA-125 level of ≥ 250 kIE/L at diagnosis. Exclusion criteria are contraindications to LMWH, ongoing or recent treatment with unfractionated heparin, LMWH, warfarin or non-vitamin K antagonist oral anticoagulants.

**Interpretation:**

This study will make an important contribution to the knowledge of the anti-neoplastic effects of tinzaparin in EOC patients and may thus guide the planning of a future study on the impact of tinzaparin on survival in EOC.

## Introduction

Ovarian cancer (OC) is the gynecological malignancy with the highest mortality rate. In Sweden, about 700 women are diagnosed with OC annually and nearly 550 succumb to the disease [1]. The majority of the OCs are of epithelial origin. Epithelial ovarian cancer (EOC) is a heterogeneous disease with different characteristics in morphology, carcinogenesis, risk factors, response to treatment, and prognosis. Many of the EOCs are assumed to originate from the fallopian tubes. Although the origin may be unclear, ovarian, fallopian tube and primary peritoneal cancers have histopathological similarities and are clinically treated similarly [2]. From a clinical perspective the EOCs, independent of origin, are considered as one entity and are usually collectively denominated as EOC.

Previous studies have shown that CA-125, a glycoprotein produced by mesoderm-derived tissues, is clinically useful as a biomarker for EOC [3], and higher pretreatment CA-125 levels have been associated with poorer progression-free and overall survival [4]. In women with advanced stage EOC receiving NACT, the magnitude of reduction in CA-125 levels has been shown to be a predictor of optimal cytoreduction during interval debulking surgery (IDS) and overall survival [5]. A decrease in CA-125 level of more than 50% after the first course of chemotherapy has indicated a better prognosis [6]. Thus, it seems that the trajectory of CA-125 might be useful as a proxy measure for the effectiveness of chemotherapy.

Pretreatment levels of several other biomarkers have also demonstrated prognostic properties in EOC. Anemia and thrombocytosis have been associated with advanced stage EOC, reduced disease-free survival and decreased overall survival [7]. Low levels of albumin predicted suboptimal debulking [8]. A high serum level of C-reactive protein (CRP), an inflammatory biomarker, was an unfavorable prognostic factor for overall survival, progression-free survival, and a predictor of postoperative residual tumor mass. Anemia and low levels of albumin and high levels of platelets and CRP may together be indicators of an activated systemic inflammatory response, indicating more advanced or generalized malignancy. High levels of interleukin 6 (IL-6), also an inflammatory biomarker, predicted reduced progression-free survival and overall survival [9]. CRP and IL-6 showed a greater decrease during NACT in patients responsive to the chemotherapy compared with those who were not responsive [10]. The impact of the trajectory of the biomarkers during chemotherapy treatment on measures of survival is unclear and merits further evaluation.

Tinzaparin (Innohep®), a low-molecular weight heparin (LMWH), is an anti-thrombotic drug used in the treatment and prevention of thromboembolism. Tinzaparin acts by facilitating the reaction between antithrombin and factor Xa, leading to inactivation of factor Xa [11]. Moreover, tinzaparin exhibits anti-inflammatory effects indicating immune system modulating properties [12], although proinflammatory effects have been reported [13]. In addition to the ability to inhibit thrombin and factor Xa, tinzaparin is effective at releasing the endothelial tissue factor (TF) pathway inhibitor, the natural inhibitor of both procoagulant and pro-neoplastic effects of TF. TF normally functions as a cellular receptor for factor VIIa and initiates the coagulation pathway. TF is present on the surface of many tumor cell types and is believed to be responsible for tumor cell procoagulant activity. Animal studies and *in vitro* studies on human OC and breast cancer cells as well as umbilical vein endothelial cells have indicated an anti-metastatic potential of tinzaparin. Various mechanisms have been suggested for the anti-metastatic effect, including upregulating the TF pathway inhibitor and E-cadherin expression and downregulating the von Willebrand factor [11]. These effects affect the metastatic process by inhibiting local invasion, the migration of tumor cells, and the promotion of tumor angiogenesis, which are all crucial to the metastatic growth and considered to be hallmarks of cancer [14]. Vascular endothelial growth factor (VEGF) produced by OC cells promotes angiogenesis in the primary tumor as well as in metastases and also increases vascular permeability resulting in ascites production [15]. In colon cancer patients, the use of tinzaparin 4500 IU daily for 30 days postoperatively reduced the levels of VEGF to the preoperative level in the first postoperative month, while VEGF remained elevated in patients who received a lower dose of tinzaparin and/or who had a shorter duration of treatment [16]. Therapeutic doses of tinzaparin have been shown to antagonize cisplatin resistance in an OC cell line by inhibiting the expression of genes that mediate cisplatin resistance [17]. A retrospective observational study of pancreatic cancer patients showed increased progression-free survival among patients receiving tinzaparin in addition to standard of care [18]. A randomized controlled trial of extended thromboprophylaxis with tinzaparin after surgery for colon cancer showed no effect on progression-free survival [19], nor did it affect progression-free or overall survival in patients treated for non-small cell lung cancer [20].

Although there are several indicators of an anti-neoplastic effect of tinzaparin in EOC, the impact of tinzaparin on disease progression and survival in EOC-patients is yet to be investigated. The aim of this study, the *Tinzaparin and Biomarkers After Neoadjuvant Treatment of Ovarian Cancer* (TABANETOC) trial is to evaluate how tinzaparin affects biomarkers that are prognostic for EOC and thus may reflect tinzaparin’s potential anti-neoplastic properties.

## Methods

### Trial design

This study is an open randomized controlled multicenter trial. The study will be conducted at three Swedish tertiary referral university hospitals and their catchment hospitals and will include women with FIGO stages III-IV EOC selected for NACT. The women scheduled for NACT will be allocated 1:1 to treatment with tinzaparin 4,500 IU/8,000 IU (weight-dependent) subcutaneously once daily (intervention group) or no tinzaparin (control group). The intervention group will start with tinzaparin on commencing the NACT. The NACT regime consists of carboplatin and paclitaxel given every third week, in accordance with the Swedish National Guidelines for Ovarian Cancer (NGOC) [21].

At inclusion (i.e. baseline), before every cycle of chemotherapy, preoperatively, and 3 weeks after the last cycle of chemotherapy, venous blood samples will be taken for measuring the biomarkers CA-125, hemoglobin, platelets, leukocytes, CRP, albumin, IL-6, VEGF, TF, D-dimer, soluble P-selectin, thrombin-antithrombin complex and thrombin generation potential. Furthermore, a panel of 92 inflammation-associated proteins will be analyzed with a highly sensitive method [22] in the plasma samples collected at baseline, preoperatively or at cycle five for subjects not undergoing surgery, and 3 weeks after the last cycle.

**Figure 1:**
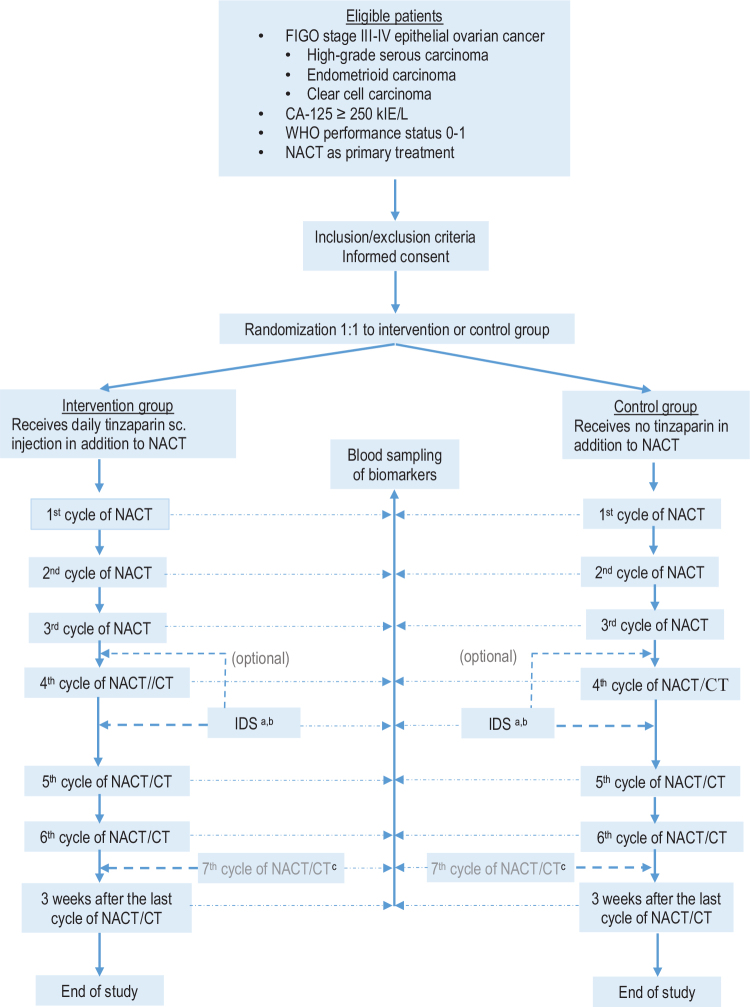
Flow chart of the TABANETOC trial. (a) All operated patients (in the treatment group as well as in the control group) will be treated with tinzaparin (Innohep®) for 28 days starting the day before surgery as clinical routine. After the 28 days of postoperative treatment they will continue with tinzaparin/no tinzaparin according to the initial allocation. (b) In some cases surgery will be performed after the third cycle of chemotherapy. (c) Some patients will receive a seventh cycle of chemotherapy. Biomarkers will be measured at that visit as well. CT: adjuvant chemotherapy; IDS: interval debulking surgery; NACT: neoadjuvant chemotherapy with a taxane/platinum doublet regimen; sc: subcutaneous.

In accordance with the NGOC [21], the patient will be evaluated clinically and by imaging diagnostics after three cycles of NACT in order to determine whether the patient should undergo IDS. After IDS, all patients will be treated with tinzaparin for 28 days as postoperative thromboprophylaxis according to clinical practice, and thereafter will continue the chemotherapy for two or three additional cycles. Participants who are allocated to tinzaparin during NACT will continue the treatment with tinzaparin during the additional two or three cycles after ending the postoperative thromboprophylaxis. The women who do not undergo IDS will continue in the trial for the following three cycles of chemotherapy with or without tinzaparin according to the original allocation. Figure 1 depicts the study design and the flow cart.

### Participants

Eligible patients are women 18 years of age or older with World Health Organization (WHO) performance status of 0–1 diagnosed with FIGO stage III-IV high-grade serous, endometrioid or clear cell EOC, and selected for NACT with a taxane/platinum doublet regime as primary treatment. The CA-125-level should be ≥ 250 kIE/L at diagnosis. Patients with ongoing or recent (within the last year) treatment with unfractionated heparin, LMWHs, warfarin or non-vitamin K antagonist oral anticoagulants or having contraindications to tinzaparin will be excluded as will pregnant patients, and patients who do not understand or speak Swedish.

### Outcomes

#### Primary outcome

The primary endpoint is the alteration in CA-125 from baseline, that is before the first course of NACT, and to each of cycles 2 through (3) 4.

#### Secondary outcomes

- The compliance to tinzaparin injections and occurrence of adverse events related to tinzaparin.
- Alterations in CA-125 after either IDS and two or three additional cycles of chemotherapy or after three or four additional cycles of NACT
- Number of objectively confirmed venous thromboembolism (VTE), that is pulmonary embolism, lower- or upper-extremity deep vein thrombosis.
- Death due to thromboembolism.

#### Secondary exploratory outcomes

A range of blood biomarkers will be measured repeatedly during the trial and the trajectory of the levels will be compared between the intervention and control group. The biomarkers are hemoglobin, leukocytes, platelets, albumin, CRP, IL-6, VEGF, TF, d-dimer, soluble P-selectin, thrombin-antithrombin complex, thrombin generation potential, and a panel of 92 inflammation-associated proteins.

### Randomization and blinding

Using the Simple Interactive Statistical Analysis software [23], the participants will be allocated in a 1:1 ratio into two groups; Group A – receiving tinzaparin (intervention group) – and Group B – control group that is without tinzaparin.

As no placebo treatment will be used the allocation will not be blinded for the participant or clinician but will be blinded for the laboratories and the researchers assessing the results.

### Statistical methods

Continuous data will be presented as mean and/or median and standard deviation, range or interquartile range, as appropriate, and categorical data as number and proportion. Univariate comparison of continuous data between groups will be conducted using analysis of variance (ANOVA), or non-parametric tests (Mann-Whitney *U*-test), as appropriate, and categorical data by means of Pearson’s chi-squared test or Fisher’s exact test, as appropriate.

To evaluate the alterations in the levels of the biomarkers over time between the intervention and control group, the measurements will be analyzed using repeated measures ANOVA models. Non-normally distributed continuous variables will be log-transformed to achieve the most appropriate transformation to a normal distribution in the analyses. The level of significance will be set at 5% (two-sided testing).

### Estimated dates for completing recruitment and presenting the results

Recruitment started in July 2022 and expected to end in December 2026. Results will be expected in 2027.

## Discussion

Cancer, inflammation and the coagulation system are closely interlinked and affect each other. Tinzaparin has the potential to interfere with these paths and alter the course of cancer disease. Although there are several indicators that tinzaparin has anti-neoplastic effects *in vitro,* these effects still need to be shown *in vivo*. This trial investigates the potential anti-neoplastic properties of tinzaparin in EOC using biomarkers as a proxy measure for neoplastic activity. The selection of CA-125 as a primary outcome is based on its widely spread use in diagnostics and follow-up in EOC, and the fact that the reduction in the level of CA-125 in patients receiving NACT is a strong predictor of surgical outcome and survival. Pretreatment levels of some of the exploratory biomarkers have in previous studies been shown to predict various important oncological outcome measures, including overall survival and progression-free survival, whereas little is known about the levels of most of the inflammatory and coagulation biomarkers, neither in patients with EOC nor about how the levels alter during chemotherapy. The intention of using a wide range of biomarkers is to capture a broad spectrum of possible effects of tinzaparin.

The TABANETOC trial has several strengths; it is a prospective randomized trial, and the main outcome measure, the level of CA-125, has a low risk of bias since it is not subjectively assessed by the investigator but analyzed in a laboratory that is blinded to the randomization assignment. This procedure may partially counteract the disadvantage of not having a placebo treatment. In addition, all analyses of CA-125 will be conducted at the same laboratory following a standardized pre-analytical handling. If significant differences are found in this trial, it would support the planning of a larger trial with clinically relevant and specific endpoints, such as progression-free and overall survival, and incidence of severe thromboembolic events. Given the severity of EOC it would be of great value if a safe and well-proven drug such as tinzaparin could provide an additional anti-neoplastic effect and even reduce occurrence of severe thromboembolic events, when given in combination with today’s standard of care treatment of EOC.

## Supplementary Material

The effect of tinzaparin on biomarkers in FIGO stages III-IV ovarian cancer patients undergoing neoadjuvant chemotherapy – the TABANETOC trial: study protocol for a randomized clinical multicenter trial

## Data Availability

The datasets used and/or analyzed during this study will be available from the corresponding author on reasonable request and in accordance with Swedish legislation.

## Trial registration

ClinicalTrials.gov (ID: NCT05284552), initial release 02/06/2022.
